# Physiological Adaptations of *Vigna radiata* to Heavy Metal Stress: Soluble Sugar Accumulation and Biomass Enhancement

**DOI:** 10.3390/plants14081191

**Published:** 2025-04-11

**Authors:** Wenjing Qi, Jinping Bai, Han Yu, Guojun Han

**Affiliations:** Department of Bioscience, Changchun Normal University, No. 677, Changji North Road, Erdao District, Changchun 130032, China

**Keywords:** *Vigna radiata*, heavy metal stress, soluble sugars, biomass enhancement, stress tolerance

## Abstract

Background: Heavy metal contamination significantly threatens crop growth and global food security. Understanding plant responses to such stress is crucial to developing stress-tolerant crops. This study explores the physiological and biochemical responses of *Vigna radiata* (L.) R. Wilczek to mercury, lead, and copper stress, focusing on the role of soluble sugar accumulation and biomass enhancement in conferring heavy metal tolerance. Methods: Commercially available *V. radiata* seeds were exposed to varying concentrations (50, 150, and 300 mg/L) of mercurous nitrate, lead nitrate, and copper chloride under controlled conditions. The germination rates, seedling growth, and physiological parameters such as the soluble sugar and protein content were analyzed using spectrophotometry and statistical methods, including ANOVA. Results: The results demonstrated that lead ion stress significantly increased the seedling dry weight, while all the tested heavy metals promoted soluble sugar accumulation. Although the heavy metals inhibited germination and growth at higher concentrations, *Vigna radiata* exhibited strong tolerance at moderate stress levels. Conclusion: This study highlights the adaptive strategies of *V. radiata*, including soluble-sugar-mediated osmotic adjustment and enhanced biomass allocation, which contribute to its resilience under heavy metal stress. These findings provide insights for breeding stress-resistant crops and managing heavy-metal-contaminated environments.

## 1. Introduction

Environmental pollution has become a major global challenge, with heavy metal pollution posing significant threats to ecosystems and human health [1,2]. Heavy metals, such as mercury, lead, and copper, are of great concern in environmental science and public health due to their persistence and high toxicity [3,4,5]. Heavy metal pollutants primarily originate from industrial emissions, agricultural activities, mining, and urban development, and they often exist as complex compounds in the environment due to their thermodynamic instability [6]. Over time, these pollutants become increasingly difficult to remediate, contaminating soil and water while bioaccumulating through the food chain, posing risks to human and ecological health [7,8,9]. Additionally, the rapid advancement of nanotechnology has exacerbated agricultural pollution, with studies indicating that nanomaterials adversely affect the plant growth, yield, and quality, ultimately threatening global food security [10].

Although soil has inherent buffering and remediation capabilities, the escalating heavy metal contamination has surpassed its self-regulatory capacity [11]. As key components of ecosystems, plants employ various protective mechanisms, such as metal chelation and reactive oxygen species (ROS) scavenging to mitigate heavy metal toxicity [12], making them a focal point in environmental pollution research. Research has shown that plant responses to heavy metal pollution involve complex physiological and molecular regulatory processes, including the absorption, accumulation, and sequestration of metal ions and the activation of antioxidant defense systems to reduce oxidative-stress-induced damage [13]. Heavy metal pollution disrupts the redox balance in plants, leading to excessive free radical production that damages cellular membranes, proteins, DNA, and other biomolecules [14]. To counteract these toxic effects, plants have evolved various physiological mechanisms, including the deployment of metal ion transporters, metal-binding proteins, and low-molecular-weight metal chelators, which effectively sequester or expel harmful metal ions to reduce toxicity [12]. Additionally, plants enhance heavy metal tolerance by accumulating soluble sugars, which regulate osmotic pressure and mitigate oxidative stress [15,16]. While many plants exhibit a certain degree of tolerance to heavy metal stress, their response mechanisms vary greatly among species, and the strategies to cope with different types of heavy metals are not entirely consistent.

*Vigna radiata* (L.) R. Wilczek, as an important economic crop, is widely cultivated globally and has become an ideal model for studying plant tolerance and adaptability mechanisms due to its short growth cycle and rich nutritional value [17,18]. Research on the physiological responses and adaptability of *V. radiata* under heavy metal stress has increased in recent years. Some studies have shown that *V. radiata* can cope with heavy metal stress through various mechanisms, such as regulating antioxidant enzyme activity, metal ion transport channels, and metabolite accumulation [19,20,21]. In heavy-metal-contaminated environments, *V. radiata* exhibits a certain degree of tolerance, particularly under low concentrations of metal pollution, where it can enhance its adaptability to environmental stress by adjusting the contents of soluble sugars, proteins, and other substances [22]. However, as the heavy metal concentrations increase, the growth of *V. radiata* is inhibited, with significant reductions in the root and shoot growth [23,24].

Despite some progress in the current research, the systematic understanding of the effects of specific heavy metals such as mercurous, lead, and copper ion stress on *V. radiata* seed germination and seedling growth remains limited [25,26]. Existing studies on the physiological and adaptive mechanisms of *V. radiata* under heavy metal stress have not fully met the needs of agricultural production and environmental protection [27]. Therefore, it is critical to further study the effects of these heavy metals on *V. radiata*’s growth and development and how *V. radiata* adjusts its physiological and metabolic processes to adapt to these environmental stresses.

This study aims to fill this research gap by systematically exploring the effects of mercurous, lead, and copper ion stress on *V. radiata* seed germination and seedling growth. This study focuses on understanding the physiological responses and adaptive mechanisms of *V. radiata* under such adverse conditions. *V. radiata* seeds and seedlings were treated with different concentrations of heavy metal ion solutions to comprehensively evaluate their effects on *V. radiata*’s germination potential, germination rate, root and shoot growth, fresh weight, dry weight, and contents of soluble sugars and proteins. Through this research, we aim to uncover how *V. radiata* adjusts its physiological and metabolic processes to adapt to heavy metal stress, thereby enhancing its tolerance and adaptability.

## 2. Results

### 2.1. Analysis of the Effects of Heavy Metal Ion Stress on V. radiata Germination

**Figure 1:**
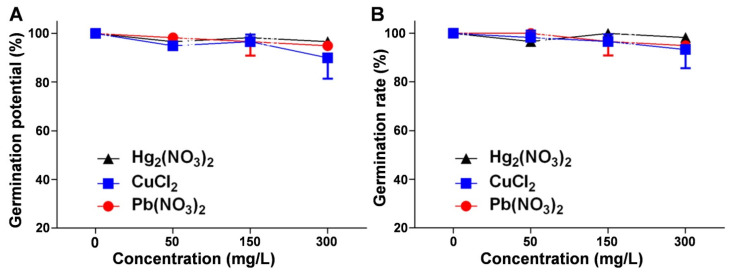
Effects of heavy metal ion stress on the germination potential and germination rate of *V. radiata* seeds: (**A**) effects of different concentrations of mercuric, copper, and lead ions on the germination potential of *V. radiata* seeds on day 3; and (**B**) effects of different concentrations of mercuric, copper, and lead ions on the germination rate of *V. radiata* seeds on day 7. Each data point represents the mean ± standard deviation from three independent experiments. Statistical significance was determined using ANOVA, with unmarked points indicating no significant difference compared to the control group.

The germination potential and germination rate, key indicators of seed vigor and germination success, were assessed under mercurous, copper, and lead ion stress. The results showed a decrease in both indicators compared to the control group, although the inhibitory effects were generally insignificant. Figure 1A demonstrates a concentration-dependent decline in the germination potential, with copper ions exerting the most pronounced inhibitory effect. Figure 1B further confirms that, except for the lead (Pb) treatment group (*p* = 0.027), the decrease in the germination rate in the mercury (Hg) treatment group (*p* = 0.296) and the Cu treatment group (*p* = 0.501) was not statistically significant (Tables S1–S3). These findings suggest that *V. radiata* seeds exhibit a degree of tolerance to heavy metal ion stress within the tested concentration range.

### 2.2. Comparative Study of the Effects of Different Heavy Metal Ion Concentrations on the Root and Shoot Growth of V. radiata Seedlings

Assessing the impact of heavy metal ion stress on *V. radiata* growth revealed significant effects on root and shoot development. At 50 mg/L mercurous ions, the *V. radiata* root length increased significantly compared to the control, suggesting a stress-induced growth response. However, at 300 mg/L, the root length decreased significantly, indicating inhibition at high concentrations.

**Figure 2:**
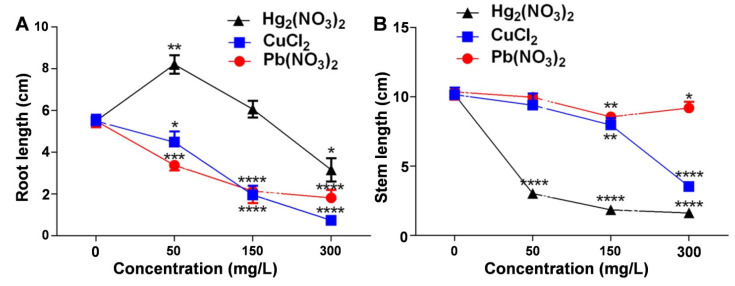
Effects of heavy metal ion stress on the root and stem growth of *V. radiata* seedlings: (**A**) effects of different concentrations of mercuric, copper, and lead ions on the root length of *V. radiata* seedlings; and (**B**) effects of different concentrations of mercuric, copper, and lead ions on the stem length of *V. radiata* seedlings. Each data point represents the mean ± standard deviation from three independent experiments. Statistical significance was determined using ANOVA, with * indicating *p* < 0.05, ** indicating *p* < 0.01, *** indicating *p* < 0.001, and **** indicating *p* < 0.0001. Unmarked points indicate no significant difference compared to the control group.

Copper and lead ions also reduced the root length in *V. radiata* seedlings, with greater inhibition at higher concentrations (Figure 2A). The shoot length measurements (Figure 2B) showed that all three heavy metal treatments inhibited shoot growth, with mercurous ions exerting the strongest effect. Low concentrations (50 mg/L) of lead and copper had minimal impact, but higher concentrations caused significant reductions, demonstrating a dose-dependent inhibitory trend.

### 2.3. Analysis of the Effects of Heavy Metal Ion Stress on the Fresh and Dry Weight of V. radiata Seedlings

**Figure 3:**
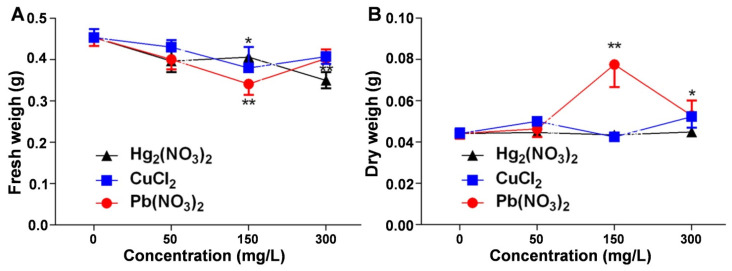
Comparison of the effects of heavy metal ion stress on the biomass of *V. radiata* seedlings: (**A**) effects of different concentrations of mercuric, copper, and lead ions on the fresh weight of *V. radiata* seedlings; and (**B**) effects of different concentrations of mercuric, copper, and lead ions on the dry weight of *V. radiata* seedlings under stress conditions. Each data point represents the mean ± standard deviation from three independent experiments. Statistical significance was determined using ANOVA, with * indicating *p* < 0.05 and ** indicating *p* < 0.01. Unmarked points indicate no significant difference compared to the control group.

The changes in the biomass reflect *V. radiata*’s adaptation to environmental stress. This study examined the effects of mercurous, copper, and lead ion stress on *V. radiata* seedling biomass, including the fresh and dry weight, to reveal the physiological responses to heavy metal pollution. Figure 3A shows that the fresh weight decreased in all the heavy metal treatments compared to the control, indicating an inhibitory effect on growth. Mercurous ions caused a dose-dependent reduction, with the strongest inhibition at 300 mg/L. Lead and copper ions also reduced the fresh weight, with a significant decline at 150 mg/L, but at higher concentrations, the fresh weight partially recovered, suggesting potential adaptive or stress-resistance mechanisms. Figure 3B indicates that mercurous and copper ions had little effect on the dry weight, whereas lead ions increased the dry weight, peaking at 150 mg/L, suggesting an activation of stress-resistance mechanisms and possibly enhancing protein synthesis. However, at excessive stress levels, growth inhibition occurred.

### 2.4. Regulatory Effects of Heavy Metal Ion Stress on Soluble Sugar and Protein Content in V. radiata Seedlings

**Figure 4:**
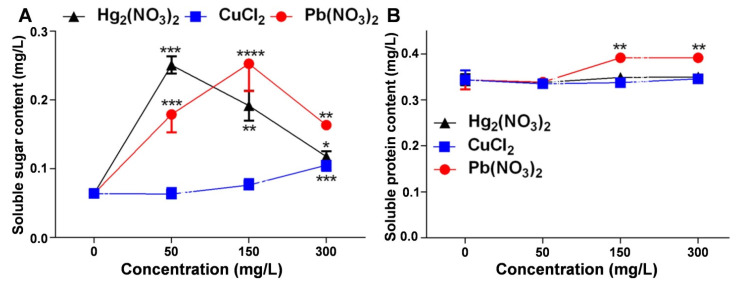
Effects of heavy metal ion stress on the physiological indices of *V. radiata* seedlings: (**A**) effects of different concentrations of mercuric, copper, and lead ions on the soluble sugar content of *V. radiata* seedlings; and (**B**) effects of different concentrations of mercuric, copper, and lead ions on the soluble protein content of *V. radiata* seedlings. Each data point represents the mean ± standard deviation from three independent experiments. Statistical significance was determined using ANOVA, with * indicating *p* < 0.05, ** indicating *p* < 0.01, *** indicating *p* < 0.001, and **** indicating *p* < 0.0001; unmarked points indicate no significant difference compared to the control group.

This study examined the changes in the soluble sugar and soluble protein content under mercurous, lead, and copper ion stress to assess their impact on the physiological functions of *V. radiata* seedlings. Figure 4A shows that the soluble sugar content in *V. radiata* seedlings increased significantly under all the heavy metal treatments. It peaked at 50 mg/L of mercurous ions and 150 mg/L of lead ions, while copper ions induced the highest accumulation at 300 mg/L (Tables S4–S6). Figure 4B indicates that mercurous and copper ions had a minimal impact on the soluble protein content, whereas lead ions significantly increased the soluble protein levels, particularly at 150 mg/L and 300 mg/L (Tables S7–S9).

### 2.5. PCA Reveals Dual Impacts of Heavy Metal Stress on Plant Morphology and Metabolism

**Figure 5:**
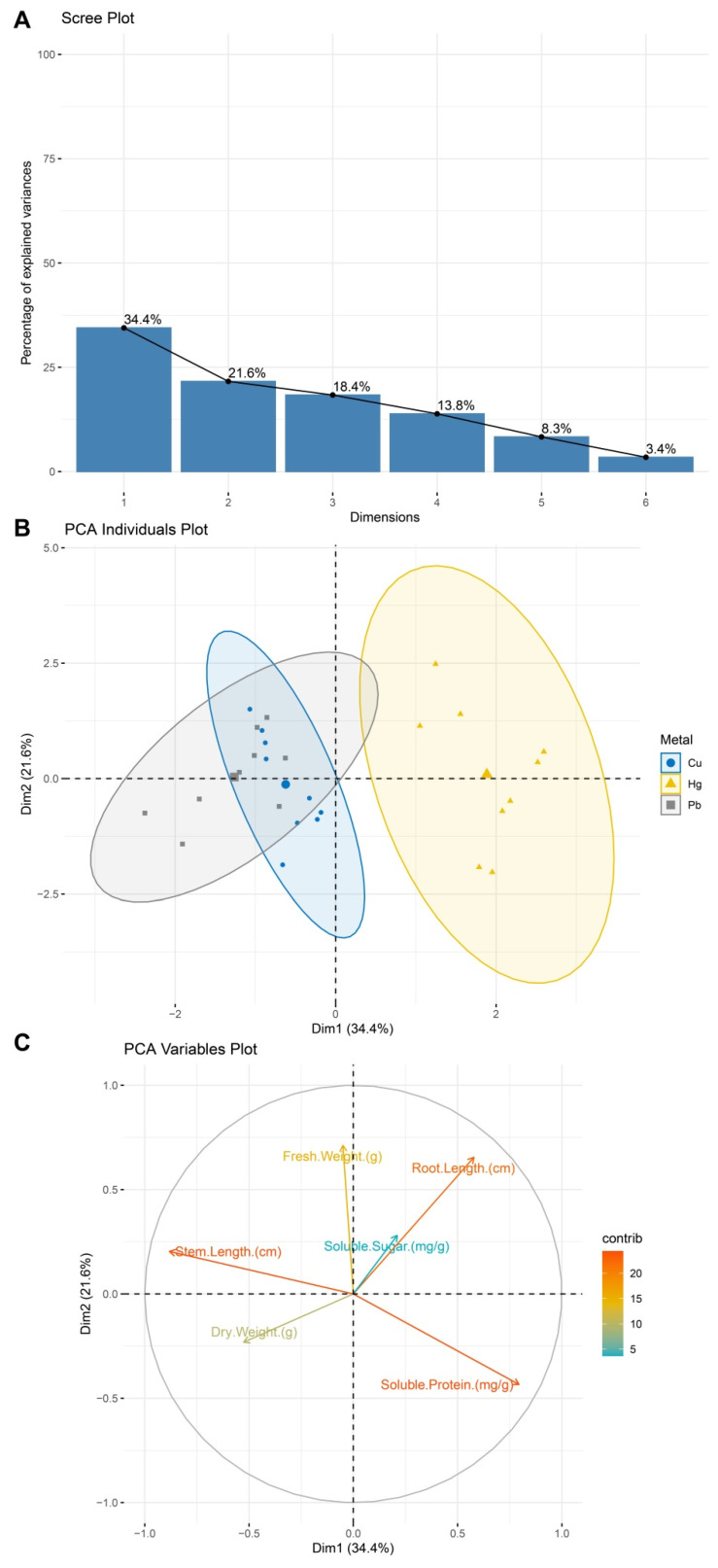
PCA results. (**A**) PCA after data standardization, showing the variance contribution rates of each principal component. (**B**) Distribution of the different heavy metal treatment groups (Cu, Hg, Pb) in the principal component space. Dots represent samples, and ellipses indicate the 95% confidence interval. Hg (yellow triangles) is positioned in the positive PC1 region, while Cu (blue circles) and Pb (gray squares) cluster in the negative PC1 region, with a partial overlap along PC2. (**C**) Contributions and directions of different variables in the principal component space, with the colors indicating the magnitudes of the contributions.

The PCA results showed that the first principal component (PC1) explained 34.4% of the variance, primarily influenced by the stem length and root length, while the second principal component (PC2) accounted for 21.6%, reflecting variations in the soluble protein and fresh weight (Figure 5A). The cumulative variance contribution of 56% effectively summarized the dataset’s key characteristics. In the PCA sample distribution plot (Figure 5B), the different heavy metal treatments exhibited distinct separations in the principal component space. The Hg-treated samples (yellow triangles) were mainly distributed in the positive PC1 region, while Cu (blue circles) and Pb (gray squares) clustered in the negative PC1 region, with a partial overlap along the negative PC2 direction, suggesting that the Cu and Pb treatments may induce similar stress effects on plant morphological and metabolic traits. The variable contribution plot (Figure 5C) further revealed that PC1 was mainly driven by the stem and root length, while PC2 was influenced by the soluble protein and fresh weight, highlighting the importance of morphological and metabolic traits in differentiating treatment groups.

## 3. Materials and Methods

### 3.1. Materials and Equipment

This study used commercially available “Tainong” *V. radiata* seeds as the experimental material. The chemical reagents used included mercurous nitrate, lead nitrate, copper chloride, glucose, bovine serum albumin (BSA), Coomassie Brilliant Blue G-250, disodium hydrogen phosphate, and sodium dihydrogen phosphate. All the chemicals were purchased from Aladdin Industrial Corporation (Los Angels, CA, USA) and were of analytical grade. The major equipment included a UV-1800 spectrophotometer (Shimadzu Corporation, Kyoto, Japan), an HH-4 digital constant temperature water bath (INESA Scientific Instrument Co., Ltd., Shanghai, China), a Centrifuge 5810 R (Eppendorf AG, Hamburg, Germany), and a Percival Scientific Model I-36VL growth chamber (Percival Scientific, Inc. Perry, IA, USA).

### 3.2. Methodology for Assessing V. radiata Responses to Different Heavy Metal Stress Concentrations

In the study of *V. radiata* seed responses to heavy metal stress, healthy, uniform-sized *V. radiata* seeds free from visible defects were carefully selected to ensure experimental consistency and reproducibility. The seeds were sterilized using 5% sodium hypochlorite solution for 5 min to remove surface microbial contaminants, followed by three thorough rinses with distilled water to eliminate residual disinfectant. Additionally, the heavy metal concentrations were determined based on commonly reported contamination levels in existing studies, considering both the environmental pollution levels and plant response patterns. To assess the specific effects of different concentrations on *V. radiata* germination and growth, we established control and experimental groups with varying concentrations, aiming to elucidate the stress effects of heavy metal ions and their concentration-dependent physiological responses [28,29,30,31]. Although mung bean seeds exhibit varying tolerance to heavy metals, this study primarily focused on exploring their physiological responses under heavy metal stress. The specific effects of different concentrations will be further investigated in future research. In 9 cm diameter Petri dishes, three layers of filter paper were placed, and 10 mL of 50 mg/L, 150 mg/L, or 300 mg/L solutions of mercurous nitrite, lead nitrate, or copper(II) chloride were added for the different heavy metal stress treatments. A control group was established under identical conditions, using an equal volume of distilled water without heavy metal addition to evaluate the effects of heavy metal stress on *V. radiata* germination and seedling growth. Each treatment and control group contained 20 surface-sterilized seeds with three replicates. The Petri dishes were placed in an incubator at 25 °C and 60% relative humidity to simulate optimal growth conditions. The experiment lasted seven days, during which the filter paper was replaced daily and the fresh metal solutions were replenished to maintain continuous exposure and ensure a clean experimental environment. On Day 7, the germinated seedlings were measured for the root length, shoot length, fresh weight, and dry weight and compared with the control group to accurately assess the impact of heavy metal stress on *V. radiata* germination and seedling growth. A detailed experimental workflow is provided in Figure S1.

### 3.3. Determination of Germination Potential and Germination Rate

To accurately assess the effects of different heavy metal ion stresses on *V. radiata* seed germination, a systematic method was employed to measure the germination potential and germination rate. *V. radiata* seeds were evenly sown at room temperature in Petri dishes containing moist, sterile sand, and each treatment group was exposed to solutions of mercurous, lead, or copper ions at varying concentrations. The germination potential and rate were determined based on the definition of germination, where seeds were considered germinated once the radicle emerged. The germination potential was calculated on the third day after sowing as the percentage of seeds germinated relative to the total number of seeds tested. Similarly, the germination rate was determined on the seventh day after sowing by calculating the cumulative percentage of germinated seeds relative to the total number of seeds tested. This method provides quantitative data on the dynamic effects of heavy metal ion stress on *V. radiata* seed germination, offering essential baseline data for further studies on the impact of heavy metal pollution on plant growth and development.

### 3.4. Determination of Soluble Sugar

In plant physiology and biochemistry studies, the soluble sugar content is a key indicator of a plant’s ability to respond to environmental changes. This study used the anthrone method to measure the soluble sugar content in *V. radiata* seedlings (Experiment 1) [32]. A 0.2% anthrone solution was prepared by dissolving anthrone in 95% sulfuric acid, mixed thoroughly, and stored in a dark place until use. A series of standard solutions of soluble sugar (glucose) were prepared to establish a calibration curve for the subsequent quantification of the sample content [33]. The procedure for determining the soluble sugar content in *V. radiata* seedlings involved the following steps: 0.5 g of fresh *V. radiata* seedlings were weighed and homogenized with 10 mL of distilled water in a pre-chilled mortar for 2–3 min until fully crushed. The homogenate was transferred to a centrifuge tube and centrifuged at 14,000 rpm and 4 °C for 15 min to remove cell debris and insoluble substances. Then, 1 mL of the supernatant was mixed with 4 mL of anthrone reagent, and the mixture was heated in a water bath at 100 °C for 5 min to complete the reaction between anthrone and soluble sugar. The mixture was then immediately cooled at room temperature for at least 5 min. Once the color reaction stabilized, the absorbance was measured at 620 nm using a spectrophotometer. The soluble sugar content in the samples was calculated using the calibration curve prepared with known concentrations.

### 3.5. Determination of Soluble Protein

The plant soluble protein content is a crucial indicator for understanding plant growth and adaptability to environmental changes. This study used the Coomassie Brilliant Blue staining method to determine the soluble protein content in *V. radiata* seedlings (Experiment 1) [34]. Coomassie Brilliant Blue G-250 staining reagent and a series of protein standard solutions of known concentrations using bovine serum albumin (BSA) were prepared. These were used to construct a standard curve for accurate protein content quantification in the subsequent samples. The procedure for determining the soluble protein content involved the following steps: 0.1 g of fresh *V. radiata* seedlings were homogenized with 1 mL of pre-prepared phosphate-buffered saline (PBS, pH 7.4) in a pre-chilled mortar for 2 min to disrupt the cellular structures and release the proteins. The homogenate was transferred to a centrifuge tube and centrifuged at 14,000 rpm and 4 °C for 10 min to separate the cell debris and insoluble components. The supernatant containing the soluble proteins was collected. Five microliters of the supernatant were mixed with 250 μL of Coomassie Brilliant Blue G-250 reagent and incubated at room temperature for 30 min. The absorbance was measured at 595 nm using a spectrophotometer. The soluble protein content in the samples was calculated using the calibration curve prepared with known protein concentrations.

### 3.6. Principal Component Analysis (PCA) and Statistical Analysis

PCA was used to integrate multiple indicator data to reveal the characteristic response patterns in relation to different metal treatments and analyze the effects of heavy metal treatments on *V. radiata*’s physiological parameters. Key growth and metabolic parameters were extracted from the treatment and control groups, including the shoot length, root length, fresh weight, dry weight, soluble protein, and soluble sugar. All the variables were standardized to eliminate scale effects. The PCA was conducted in R using the FactoMineR package, extracting the first principal components (PC1 and PC2) to analyze the variance contribution and variable loadings. The cumulative variance reached 56%, making it suitable for summarizing the major physiological differences. For the statistical significance analysis, GraphPad Prism version 6.0 was used. ANOVA was applied to determine the significance levels, with the *p*-values interpreted as follows. *p* < 0.05 was considered statistically significant; *p* < 0.01, *p* < 0.001, and *p* < 0.0001 indicated progressively higher significance levels. Unmarked results indicated no statistically significant difference compared with the control group.

### 3.7. Statistical Analysis

To ensure the reliability and accuracy of the study results, GraphPad Prism 6 software was used for the statistical significance analysis. The criteria for *p*-value significance were as follows: * indicates *p* < 0.05, denoting statistical significance; ** indicates *p* < 0.01, denoting higher significance; *** indicates *p* < 0.001; **** indicates *p* < 0.0001, indicating progressively higher levels of statistical significance.

## 4. Discussion

This study systematically evaluated the effects of mercurous, lead, and copper ion stress on *V. radiata* seed germination and seedling growth. The results indicate that while heavy metal stress affected the germination potential and germination rate of *V. radiata*, the overall impact was insignificant. This finding aligns with previous studies in other plant species, such as rice and wheat, which also exhibited strong tolerance to low concentrations of heavy metal stress [35,36]. However, unlike these studies, we observed a significant concentration-dependent inhibitory effect of heavy metal stress on the root and shoot growth in *V. radiata*, particularly under the mercurous and copper ion treatments, where the inhibition was more pronounced at higher concentrations. This provides new insights into *V. radiata*’s sensitivity to specific heavy metal stress. Heavy metal stress also significantly altered the soluble sugar and protein content, highlighting the osmotic regulatory mechanisms involved in plant adaptation to metal stress. This study reveals the complexity of plant physiological responses and offers theoretical support for breeding stress-resistant crops and developing phytoremediation strategies for contaminated soils. However, the specific mechanisms underlying heavy metal stress tolerance remain complex and warrant further investigation.

The effects of heavy metal stress on the germination potential and germination rate of *V. radiata* were relatively mild, with copper ions exhibiting a more pronounced inhibitory effect on the germination potential at higher concentrations. Studies suggest that seed germination is closely linked to the plant’s antioxidant system, as heavy metal contamination can activate antioxidant defense mechanisms and influence physiological processes [37]. For instance, research indicates that cadmium accumulation in barley roots leads to oxidative stress via carboxylation of the SOD, CAT, GR, and GSTS proteins [38]. However, *V. radiata* demonstrated strong adaptability at low heavy metal concentrations, similar to the physiological resilience observed in other species under environmental stress. Studies have shown that *Zea mays* exhibits organ-specific responses in its roots and leaves under different types of heavy metal stress, whereas *Vetiveria zizanioides* can tolerate copper stress up to 400 mg/kg and displays optimal root development at 200 mg/kg [39,40]. Some studies suggest that plants secrete antioxidants to mitigate heavy metal stress, supporting the hypothesis that *V. radiata* may employ intrinsic defense mechanisms during germination, such as enhancing membrane-associated antioxidant defenses. Given that plants counteract heavy metal stress by enhancing their antioxidant capacity, future research should explore whether heavy metal stress induces ROS production [41,42].

Heavy metal stress exhibited concentration-dependent inhibitory effects on the root and shoot growth of *V. radiata* seedlings, with mercurous and copper ions causing significant inhibition of the root growth at higher concentrations. Similarly, previous studies have shown that heavy metals not only directly affect plant growth but also inhibit it by disrupting the cell wall and water transport systems of plants [43]. These effects are likely due to the direct toxicity of metal ions to plant roots, especially when high metal concentrations in soil solutions reduce the ability of roots to absorb water and nutrients [44]. However, lead ions increased the dry weight of *V. radiata* seedlings at certain concentrations, suggesting an adaptive response. This effect may result from enhanced metabolic activity, leading to greater biomass accumulation. It is further speculated that this promotion may involve stress signaling, osmotic regulation, and antioxidant defense mechanisms, ultimately contributing to the increased dry weight [45,46,47].

Additionally, accumulating soluble sugars under heavy metal stress is a critical adaptive response in plants. Significant increases in the soluble sugar content were observed in *V. radiata* seedlings under mercurous, lead, and copper ion stress. This suggests plants may regulate osmotic adjustment mechanisms to adapt to adverse conditions [48]. These changes help plants maintain the osmotic balance under heavy metal stress and may enhance the antioxidant capacity, mitigating heavy metal toxicity. Soluble sugars play a vital role in osmotic adjustment and oxidative stress tolerance by maintaining cellular redox homeostasis and reducing membrane lipid peroxidation under abiotic stress [49,50]. Similar findings suggest that Zn, Ni, Cd and Cu heavy metal stress significantly affects plant biomass accumulation and root development, with oxidative stress regulation playing a key role in tolerance mechanisms [39]. Further studies indicate that plants adapt to heavy metal stress by modulating sugar metabolism, reducing oxidative damage, and maintaining redox homeostasis [40,45]. This mechanism may explain *V. radiata*’s strong adaptability to heavy metal stress, as sugars act as osmoprotectants, stabilizing cellular function under adverse conditions. Regarding the genetic regulatory mechanisms of heavy metal stress, while this study did not delve into specific gene expression changes, previous research highlights the critical role of genetic regulation in plant tolerance to heavy metals [51]. Genes associated with metal transport, detoxification, and antioxidant mechanisms are often activated under heavy metal stress, helping plants reduce endogenous metal accumulation and enhance stress tolerance [51,52,53]. Studies suggest that specific transcription factors regulate key metabolic pathways in response to heavy metal stress, including metal ion uptake, storage, and efflux [54]. Future research should explore the genetic mechanisms underlying these metabolic adaptations and their role in heavy metal stress tolerance.

Despite advancing our understanding of *V. radiata*’s response mechanisms to heavy metal stress, this study has several limitations. First, it primarily focuses on seed germination and early seedling growth, lacking insights into the effects on mature plants and long-term stress responses. Second, while the soluble sugar and protein content were analyzed, the molecular mechanisms underlying heavy metal stress tolerance, including the gene expression changes and signaling pathways, remain unclear. Additionally, no images of *V. radiata* seedlings or seeds under heavy metal stress were provided, limiting the visual assessment of the root, leaf, and seed morphology. Furthermore, this study did not consider the bioavailability of heavy metals in soil or the differences in root uptake efficiency, which may affect the real-world applicability of the findings. The short experimental duration also prevented a comprehensive evaluation of metal transport. Future studies should quantify the metal accumulation in different plant tissues to assess how *V. radiata* mitigates heavy metal toxicity through exclusion or translocation mechanisms.

Future research should address these limitations and expand this study’s scope. Long-term experiments on mature plants are needed to understand the heavy metal stress effects throughout the plant’s life cycle. Investigating the physiological and molecular responses at different developmental stages will provide a more comprehensive view of the stress adaptation mechanisms. Additionally, integrating transcriptomics and proteomics will help elucidate the gene regulation and signaling pathways involved in heavy metal tolerance. Considering practical applications, future studies should explore multi-metal stress conditions and assess their impact on the crop yield and quality. Finally, since field conditions involve complex interactions between heavy metals, nutrients, and environmental factors, future studies should simulate real-world agricultural environments to evaluate *V. radiata*’s adaptability beyond controlled conditions.

## 5. Conclusions

This study provides a preliminary assessment of mercurous, lead, and copper ion stress on *V. radiata* seed germination and seedling growth, estimating their physiological impact. The results indicate that while heavy metal stress affected the germination potential and germination rate, the impact was insignificant, suggesting that *V. radiata* exhibits tolerance to 50–300 mg/L of heavy metal nitrates and chlorides. The primary effects were observed in the seedlings, with *V. radiata* showing greater tolerance to lead ions than other metals. Notably, low mercurous ion concentrations (50 mg/L) promoted root elongation. Heavy metal stress significantly increased the seedling dry weight and altered the biomass allocation, particularly under 150 mg/L lead ion stress, which strongly enhanced the dry weight *(Graphic abstract)*. Further analysis revealed that heavy metal stress promoted soluble protein accumulation, although the dry weight increase was more likely due to enhanced soluble sugar accumulation. These findings reveal *V. radiata*’s adaptive and physiological responses to heavy metal stress, offering new insights into plant resilience under heavy metal contamination.

## Data Availability

The data generated or analyzed in this study are available from the corresponding authors upon reasonable request.

## Supplementary Materials

The following supporting information can be downloaded at https://www.mdpi.com/article/10.3390/plants14081191/s1, Figure S1: Research workflow for the analysis of *V. radiata* seed germination and physiological indices under heavy metal stress. Table S1: Homogeneity of variance test for the germination rate. Table S2: ANOVA results for the germination rate. Table S3: Multiple comparisons of the germination rate. Table S4: Homogeneity of variance test for the soluble sugar content. Table S5: ANOVA results for the soluble sugar content. Table S6: Post hoc test results for the soluble sugar content. Table S7: Homogeneity of variance test for the soluble protein content. Table S8: ANOVA results for the soluble protein content. Table S9: Post hoc test results for the soluble protein content.

## Abbreviations

ANOVA	Analysis of variance
PCA	Principal component analysis
