# Supplementary material for: Physiological Adaptations of Vigna radiata to Heavy Metal Stress: Soluble Sugar Accumulation and Biomass Enhancement
Source: Plants (Basel). 2025 Apr 11;14(8):1191. doi: 10.3390/plants14081191 (PMC12030698; doi:10.3390/plants14081191)
Supplement: Supplementary file 1 [file plants-14-01191-s001.zip › plants-3440583-supplementary.pdf]

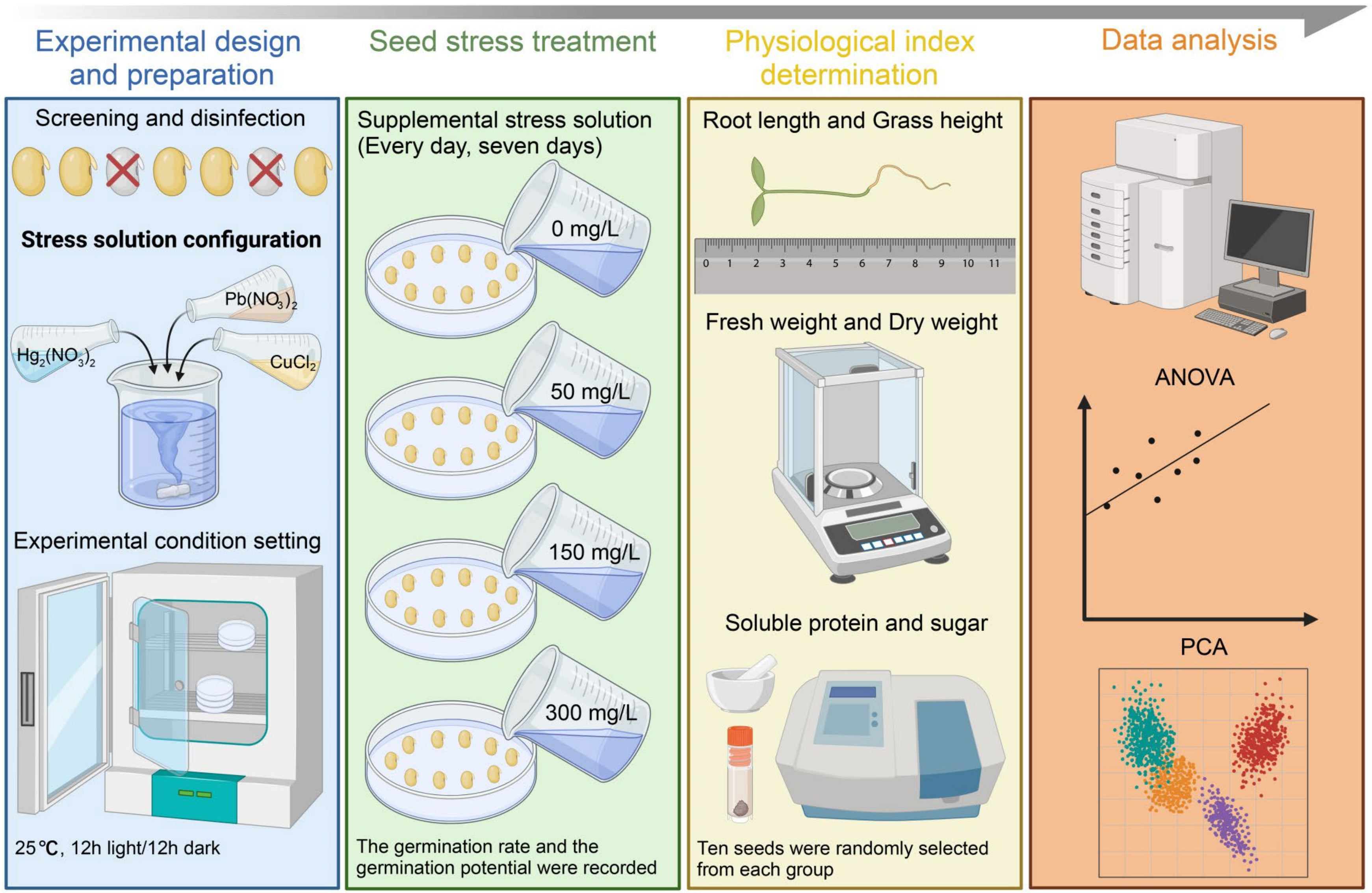

Figure S1. Research Workflow for the Analysis of *V. radiata* Seed Germination and Physiological Indices Under Heavy Metal Stress.

Table S1: Homogeneity of Variance Test for Germination Rate.

Germination Rate	Test Type	Levene Statistic	df1	df2	Significance (*p*-value)
**Hg Germination Rate**	Based on Mean	8.000	2	6	0.020
	Based on Median	0.500	2	6	0.630
	Based on Median with Adjusted df	0.500	2	4.000	0.640
	Based on Trimmed Mean	6.301	2	6	0.034
**Pb Germination Rate**	Based on Mean	16.000	2	6	0.004
	Based on Median	1.000	2	6	0.422
	Based on Median with Adjusted df	1.000	2	2.000	0.500
	Based on Trimmed Mean	12.602	2	6	0.007
**Cu Germination Rate**	Based on Mean	2.400	2	6	0.171
	Based on Median	0.800	2	6	0.492
	Based on Median with Adjusted df	0.800	2	4.545	0.504
	Based on Trimmed Mean	2.245	2	6	0.187

Table S2: ANOVA Results for Germination Rate.

Germination Rate	Source of Variation	Sum of Squares	df	Mean Square	*F*	Significance (*p*-value)
**Hg Germination Rate**	Between Groups	0.002	2	0.001	1.500	0.296
	Within Groups	0.003	6	0.001		
	Total	0.005	8			
**Pb Germination Rate**	Between Groups	0.004	2	0.002	7.000	0.027
	Within Groups	0.002	6	0.000		
	Total	0.006	8			
**Cu Germination Rate**	Between Groups	0.004	2	0.002	0.778	0.501
	Within Groups	0.015	6	0.003		
	Total	0.019	8			

Table S3: Multiple Comparisons of Germination Rate.

Dependent Variable	Method	Concentration (I)	Concentration (J)	Mean Difference (I-J)	Std. Error	Significance (*p*-value)	Lower Bound	Upper Bound
**Hg Germination Rate**	Bonferroni	50.00	150.00	-0.03333	0.01925	0.402	-0.0966	0.0299
		50.00	300.00	-0.01667	0.01925	1.000	-0.0799	0.0466
		150.00	50.00	0.03333	0.01925	0.402	-0.0299	0.0966
		150.00	300.00	0.01667	0.01925	1.000	-0.0466	0.0799
		300.00	50.00	0.01667	0.01925	1.000	-0.0466	0.0799
		300.00	150.00	-0.01667	0.01925	1.000	-0.0799	0.0466
	Games-Howell	50.00	150.00	-0.03333	0.01667	0.313	-0.1315	0.0648
		50.00	300.00	-0.01667	0.02357	0.773	-0.1007	0.0673
		150.00	50.00	0.03333	0.01667	0.313	-0.0648	0.1315
		150.00	300.00	0.01667	0.01667	0.645	-0.0815	0.1148
		300.00	50.00	0.01667	0.02357	0.773	-0.0673	0.1007
		300.00	150.00	-0.01667	0.01667	0.645	-0.1148	0.0815
**Pb Germination Rate**	Bonferroni	50.00	150.00	0.01667	0.01361	0.800	-0.0281	0.0614

Dependent Variable	Method	Concentration (I)	Concentration (J)	Mean Difference (I-J)	Std. Error	Significance (*p*-value)	Lower Bound	Upper Bound
		50.00	300.00	**0.05000**	0.01361	**0.031**	**0.0053**	**0.0947**
		150.00	50.00	-0.01667	0.01361	0.800	-0.0614	0.0281
		150.00	300.00	0.03333	0.01361	0.149	-0.0114	0.0781
		300.00	50.00	**-0.05000**	0.01361	**0.031**	**-0.0947**	**-0.0053**
		300.00	150.00	-0.03333	0.01361	0.149	-0.0781	0.0114
	Games-Howell	50.00	150.00	0.01667	0.01667	0.645	-0.0815	0.1148
		50.00	300.00	0.05000	0.00000	0.000	0.0500	0.0500
		150.00	50.00	-0.01667	0.01667	0.645	-0.1148	0.0815
		150.00	300.00	0.03333	0.01667	0.313	-0.0648	0.1315
		300.00	50.00	-0.05000	0.00000	0.000	-0.0500	-0.0500
		300.00	150.00	-0.03333	0.01667	0.313	-0.1315	0.0648
**Cu Germination Rate**	Bonferroni	50.00	150.00	0.01667	0.04082	1.000	-0.1175	0.1509
		50.00	300.00	0.05000	0.04082	0.800	-0.0842	0.1842
		150.00	50.00	-0.01667	0.04082	1.000	-0.1509	0.1175
		150.00	300.00	0.03333	0.04082	1.000	-0.1009	0.1675

Dependent Variable	Method	Concentration (I)	Concentration (J)	Mean Difference (I-J)	Std. Error	Significance (*p*-value)	Lower Bound	Upper Bound
		300.00	50.00	-0.05000	0.04082	0.800	-0.1842	0.0842
		300.00	150.00	-0.03333	0.04082	1.000	-0.1675	0.1009
	Games-Howell	50.00	150.00	0.01667	0.02357	0.773	-0.0673	0.1007
		50.00	300.00	0.05000	0.04714	0.604	-0.1709	0.2709
		150.00	50.00	-0.01667	0.02357	0.773	-0.1007	0.0673
		150.00	300.00	0.03333	0.04714	0.779	-0.1875	0.2542
		300.00	50.00	-0.05000	0.04714	0.604	-0.2709	0.1709
		300.00	150.00	-0.03333	0.04714	0.779	-0.2542	0.1875

Table S4: Homogeneity of Variance Test for Soluble Sugar Content.

Soluble Sugar	Test Type	Levene Statistic	df1	df2	Significance (*p*-value)
**Cu**	Based on Mean	0.000	2	6	1.000
	Based on Median	0.000	2	6	1.000
	Based on Median with Adjusted df	0.000	2	6.000	1.000
	Based on Trimmed Mean	0.000	2	6	1.000
**Hg**	Based on Mean	9.201	2	6	0.015
	Based on Median	0.701	2	6	0.533
	Based on Median with Adjusted df	0.701	2	2.301	0.578
	Based on Trimmed Mean	7.442	2	6	0.024
**Pb**	Based on Mean	8.326	2	6	0.019
	Based on Median	0.678	2	6	0.542
	Based on Median with Adjusted df	0.678	2	3.296	0.567
	Based on Trimmed Mean	6.778	2	6	0.029

Table S5: ANOVA Results for Soluble Sugar Content.

Soluble Sugar	Source of Variation	Sum of Squares	df	Mean Square	*F*	Significance (*p*-value)
**Cu**	Between Groups	7154.640	2	3577.320	18.946	0.003
	Within Groups	1132.880	6	188.813		
	Total	8287.520	8			
**Hg**	Between Groups	17450.569	2	8725.284	0.363	0.710
	Within Groups	144238.960	6	24039.827		
	Total	161689.529	8			
**Pb**	Between Groups	20617.280	2	10308.640	0.579	0.589
	Within Groups	106772.640	6	17795.440		
	Total	127389.920	8			

Table S6: Post Hoc Test Results for Soluble Sugar Content.

Dependent Variable	Method	Concentration (I)	Concentration (J)	Mean Difference (I-J)	Std. Error	Significance (*p*-value)	Lower Bound	Upper Bound
**Cu**	Bonferroni	50.00	150.00	-19.80000	11.21943	0.384	-56.6834	17.0834
		50.00	300.00	**-67.20000**	11.21943	**0.003**	**-104.0834**	**-30.3166**
		150.00	50.00	19.80000	11.21943	0.384	-17.0834	56.6834
		150.00	300.00	**-47.40000**	11.21943	**0.017**	**-84.2834**	**-10.5166**
		300.00	50.00	**67.20000**	11.21943	**0.003**	**30.3166**	**104.0834**
		300.00	150.00	**47.40000**	11.21943	**0.017**	**10.5166**	**84.2834**
	Games-Howell	50.00	150.00	-19.80000	11.21943	0.291	-59.7859	20.1859
		50.00	300.00	**-67.20000**	11.21943	**0.009**	**-107.1859**	**-27.2141**
		150.00	50.00	19.80000	11.21943	0.291	-20.1859	59.7859
		150.00	300.00	**-47.40000**	11.21943	**0.029**	**-87.3859**	**-7.4141**
		300.00	50.00	**67.20000**	11.21943	**0.009**	**27.2141**	**107.1859**
		300.00	150.00	**47.40000**	11.21943	**0.029**	**7.4141**	**87.3859**
**Hg**	Bonferroni	50.00	150.00	105.00000	126.59602	1.000	-311.1787	521.1787
		50.00	300.00	73.86667	126.59602	1.000	-342.3121	490.0454
		150.00	50.00	-105.00000	126.59602	1.000	-521.1787	311.1787

Dependent Variable	Method	Concentration (I)	Concentration (J)	Mean Difference (I-J)	Std. Error	Significance (p-value)	Lower Bound	Upper Bound
		150.00	300.00	-31.13333	126.59602	1.000	-447.3121	385.0454
		300.00	50.00	-73.86667	126.59602	1.000	-490.0454	342.3121
		300.00	150.00	31.13333	126.59602	1.000	-385.0454	447.3121
	Games-Howell	50.00	150.00	105.00000	45.15819	0.198	-86.4709	296.4709
		50.00	300.00	73.86667	149.68610	0.881	-774.9745	922.7078
		150.00	50.00	-105.00000	45.15819	0.198	-296.4709	86.4709
		150.00	300.00	-31.13333	153.73504	0.978	-821.3768	759.1101
		300.00	50.00	-73.86667	149.68610	0.881	-922.7078	774.9745
		300.00	150.00	31.13333	153.73504	0.978	-759.1101	821.3768
**Pb**	Bonferroni	50.00	150.00	78.00000	108.92028	1.000	-280.0705	436.0705
		50.00	300.00	114.80000	108.92028	0.997	-243.2705	472.8705
		150.00	50.00	-78.00000	108.92028	1.000	-436.0705	280.0705
		150.00	300.00	36.80000	108.92028	1.000	-321.2705	394.8705
		300.00	50.00	-114.80000	108.92028	0.997	-472.8705	243.2705
		300.00	150.00	-36.80000	108.92028	1.000	-394.8705	321.2705
	Games-Howell	50.00	150.00	78.00000	133.39594	0.837	-443.1730	599.1730

Dependent Variable	Method	Concentration (I)	Concentration (J)	Mean Difference (I-J)	Std. Error	Significance (*p*-value)	Lower Bound	Upper Bound
		50.00	300.00	114.80000	113.24620	0.639	-552.1978	781.7978
		150.00	50.00	-78.00000	133.39594	0.837	-599.1730	443.1730
		150.00	300.00	36.80000	70.51030	0.870	-378.3857	451.9857
		300.00	50.00	-114.80000	113.24620	0.639	-781.7978	552.1978
		300.00	150.00	-36.80000	70.51030	0.870	-451.9857	378.3857

Table S7: Homogeneity of Variance Test for Soluble Protein Content.

Soluble Protein	Test Type	Levene Statistic	df1	df2	Significance (*p*-value)
**Hg**	Based on Mean	1.556	2	6	0.286
	Based on Median	0.375	2	6	0.702
	Based on Median with Adjusted df	0.375	2	4.923	0.705
	Based on Trimmed Mean	1.413	2	6	0.314
**Cu**	Based on Mean	0.000	2	6	1.000
	Based on Median	0.000	2	6	1.000
	Based on Median with Adjusted df	0.000	2	6.000	1.000
	Based on Trimmed Mean	0.000	2	6	1.000
**Pb**	Based on Mean	**12.285**	2	6	**0.008**
	Based on Median	0.970	2	6	0.431
	Based on Median with Adjusted df	0.970	2	2.037	0.506
	Based on Trimmed Mean	**10.013**	2	6	**0.012**

Table S8: ANOVA Results for Soluble Protein Content.

Soluble Protein	Source of Variation	Sum of Squares	df	Mean Square	*F*	Significance (*p*-value)
**Hg**	Between Groups	0.000	2	0.000	**5109.085**	**0.000**
	Within Groups	0.000	6	0.000		
	Total	0.000	8			
**Cu**	Between Groups	0.000	2	0.000	**15084.336**	**0.000**
	Within Groups	0.000	6	0.000		
	Total	0.000	8			
**Pb**	Between Groups	0.006	2	0.003	**627.046**	**0.000**
	Within Groups	0.000	6	0.000		
	Total	0.006	8			

Table S9: Post Hoc Test Results for Soluble Protein Content.

Dependent Variable	Method	Concentration (I)	Concentration (J)	Mean Difference (I-J)	Std. Error	Significance (*p*-value)	Lower Bound	Upper Bound
**Hg**	Bonferroni	50.00	150.00	**-0.01088**	0.00013	**0.000**	**-0.0113**	**-0.0104**
		50.00	300.00	**-0.01214**	0.00013	**0.000**	**-0.0126**	**-0.0117**
		150.00	50.00	**0.01088**	0.00013	**0.000**	**0.0104**	**0.0113**
		150.00	300.00	**-0.00126**	0.00013	**0.000**	**-0.0017**	**-0.0008**
		300.00	50.00	**0.01214**	0.00013	**0.000**	**0.0117**	**0.0126**
		300.00	150.00	**0.00126**	0.00013	**0.000**	**0.0008**	**0.0017**
	Games-Howell	50.00	150.00	**-0.01088**	0.00010	**0.000**	**-0.0113**	**-0.0104**
		50.00	300.00	**-0.01214**	0.00013	**0.000**	**-0.0128**	**-0.0115**
		150.00	50.00	**0.01088**	0.00010	**0.000**	**0.0104**	**0.0113**
		150.00	300.00	**-0.00126**	0.00015	**0.004**	**-0.0018**	**-0.0007**
		300.00	50.00	**0.01214**	0.00013	**0.000**	**0.0115**	**0.0128**
		300.00	150.00	**0.00126**	0.00015	**0.004**	**0.0007**	**0.0018**
**Cu**	Bonferroni	50.00	150.00	**-0.00275**	0.00007	**0.000**	**-0.0030**	**-0.0025**
		50.00	300.00	**-0.01102**	0.00007	**0.000**	**-0.0112**	**-0.0108**
		150.00	50.00	**0.00275**	0.00007	**0.000**	**0.0025**	**0.0030**

**Dependent Variable**	**Method**	**Concentration (I)**	**Concentration (J)**	**Mean Difference (I-J)**	**Std. Error**	**Significance (*p*-value)**	**Lower Bound**	**Upper Bound**
		150.00	300.00	**-0.00826**	0.00007	**0.000**	**-0.0085**	**-0.0080**
		300.00	50.00	**0.01102**	0.00007	**0.000**	**0.0108**	**0.0112**
		300.00	150.00	**0.00826**	0.00007	**0.000**	**0.0080**	**0.0085**
**Pb**	Bonferroni	50.00	150.00	**-0.05257**	0.00172	**0.000**	**-0.0582**	**-0.0469**
		50.00	300.00	**-0.05280**	0.00172	**0.000**	**-0.0584**	**-0.0472**
		150.00	50.00	**0.05257**	0.00172	**0.000**	**0.0469**	**0.0582**
		150.00	300.00	-0.00023	0.00172	1.000	-0.0059	0.0054
		300.00	50.00	**0.05280**	0.00172	**0.000**	**0.0472**	**0.0584**
		300.00	150.00	0.00023	0.00172	1.000	-0.0054	0.0059
